# Stereotactic Radiofrequency Ablation of an Unresectable Intrahepatic Cholangiocarcinoma (ICC): Transforming an Aggressive Disease into a Chronic Condition

**DOI:** 10.1007/s00270-020-02443-3

**Published:** 2020-03-11

**Authors:** Gregor Laimer, Nikolai Jaschke, Maximilian Gottardis, Peter Schullian, Daniel Putzer, Wolfgang Sturm, Reto Bale

**Affiliations:** 1 Interventional Oncology-Microinvasive Therapy (SIP), Department of Radiology, Medical University Innsbruck, Anichstr. 35, 6020 Innsbruck, Austria; 2 Department of Internal Medicine I, Gastroenterology, Hepatology, Endocrinology and Metabolism, Medical University Innsbruck, Anichstr. 35, 6020 Innsbruck, Austria

**Keywords:** Stereotactic radiofrequency ablation, Cholangiocarcinoma, Navigation, Liver, Stereotactic irreversible electroporation, Inferior vena cava syndrome

## Abstract

In 2010, we reported on a 72-year-old patient with a large, unresectable cholangiocarcinoma with intrahepatic metastases, which was treated by stereotactic radiofrequency ablation (SRFA) in three consecutive sessions. Within the last nine years, the same patient has received seven additional ablation sessions for a total of ten recurrent intrahepatic lesions. One year after the last SRFA, the patient’s liver function is still within the physiological range, suggesting that this approach is not only sufficient for locally controlling tumor disease, but also for sparing healthy tissue. Moreover, periods of hospitalization were relatively short, while procedure-related pain was generally mild. In summary, SRFA has turned an aggressive disease with a devastating prognosis into a chronic condition while improving the patient’s quality of life.

## Introduction

Radiofrequency ablation (RFA) has emerged as a minimally invasive, potentially curative approach for the treatment of liver tumors. Due to the creation of overlapping ablation zones using multiple needle approaches with 3D treatment planning and stereotactic needle placement (thus termed “stereotactic RFA” or “SRFA”), the spectrum of locally curable liver lesions can be substantially increased [1–3].

We have previously reported on a Caucasian male who underwent three consecutive SRFA treatments within 27 months for a large, unresectable intrahepatic cholangiocarcinoma (max. diameter of 10 cm), a satellite tumor (4 cm) and two distant liver recurrences, while preserving his liver function and significantly prolonging his survival [1]. Here, we further expand this initial report by the following seven SRFA treatments within the last nine years.

## Case Report

**Fig. 1:**
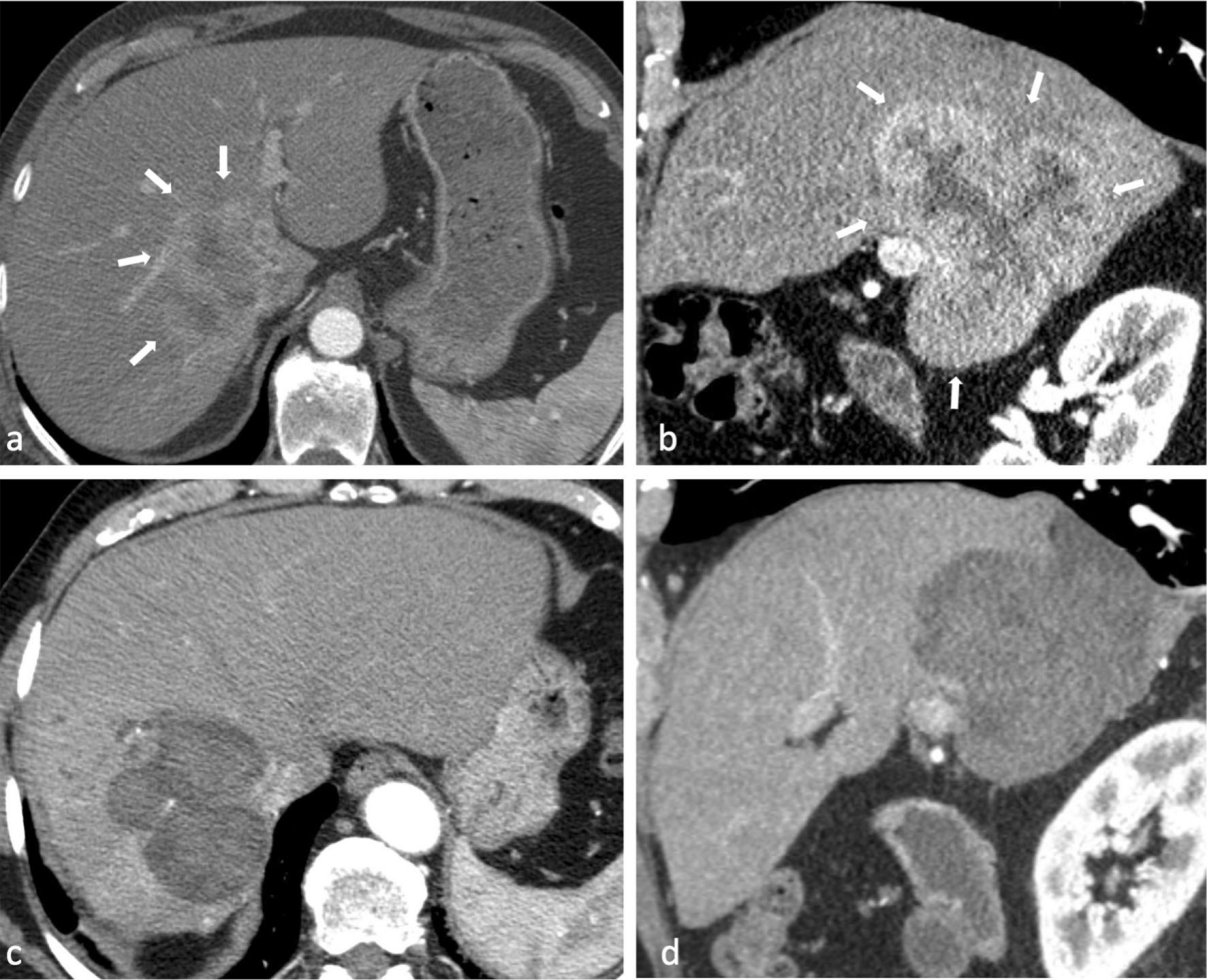
A,B Axial (**A)** and sagittal (**B)** plane of contrast-enhanced CT scan showing initial tumor in liver segments I, V, VI, VII, VIII with 10 cm in diameter (white arrows). **C, D** Axial (**C)** and sagittal (**D)** plane of contrast-enhanced CT scan 27 months after the first SRFA (prior to third SRFA session of two distant recurrent nodules) with no residual tumor or local recurrence of initial tumor(s)

In 2007 and 2009, a 72-year-old male exhibiting a large, histopathologically verified intrahepatic cholangiocarcinoma (ICC) with a maximum diameter of 10 cm involving liver segments I, V, VI, VII, VIII (Fig. 1A, B), as well as a satellite tumor (4 cm), was treated with SRFA at our institution. These first three ablation sessions, as well as the exact technical procedure of SRFA itself, have already been reported in detail elsewhere [1].

**Fig. 2:**
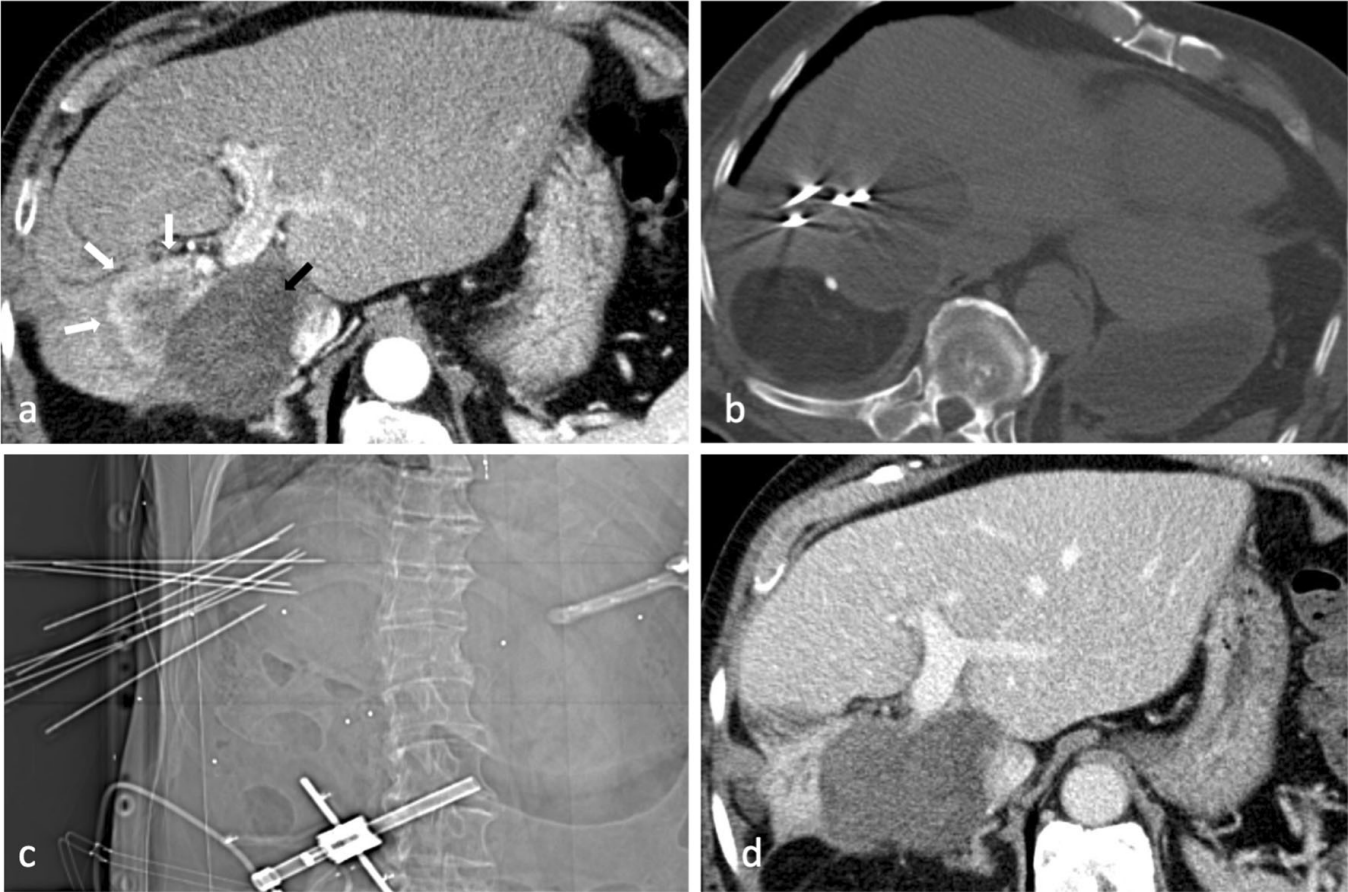
A Arterial phase CT showing local recurrence with 4.5 cm in diameter (white arrow) directly adjacent to large necrosis zone (black arrow) in November 2012. **B, C** Axial non-enhanced control CT and scout view, (**C**) with nine coaxial needles in place during the fourth SRFA session in December 2012. **D** First follow-up with no evidence of residual/recurrent tumor 4 months later (March 2013)

In November 2012, 67 months after the first SRFA, a routinely performed follow-up CT scan revealed local tumor recurrence located centrally in liver segment V (Fig. 2A). Subsequently, the fourth SRFA session was performed in December 2012 without any complications (Fig. 2B, C). In the following six years, the patient underwent six additional SRFA treatments up until March 2018. Table 1 summarizes the different sessions including technical and clinical details. All sessions, including stereotactic irreversible electroporation (SIRE), were performed with the same optical-based stereotactic navigation system (StealthStation Treon plus, Medtronic Inc., Louisville, KY, USA).

**Table 1:** Overview of ablation sessions

Date	Nr. ablated lesions	Max. diameter (mm)	Nr. of coaxial needles	Classification	Liver segment	Location	Time of ablation (min)	Complication	Length of hospital stay (days)	Treatment of complication	Additional therapy
02.05.07	1	100	18	Initial	I, V, VI, VII, VIII	Vessel, bile duct, subphrenic	300	Post-ablation syndrome	21	–	
13.06.07	2	40/12	6/1	Initial/residual	VII/V	Subcapsular/VCI	44	Pleural effusion	6	Conservative	
09.07.09	2	10/5	2/1	New/new	IVb/VIII,IVa	Subcapsular/subcapsular	36	None	3	–	Ablation of lower mediastinal lymph node (1 needle)
07.12.12	1	45	9	Recurrence	V	Central	44	None	4	–	
22.05.13	1	10	2	New	IVb	Subcapsular	6	None	3	–	
27.02.15	2 (1)	28/28	2/3	Recurrence/recurrence	IVa/V,VIII	Subphrenic/gallbladder	20	Pleural effusion	15	Drainage	SIRE of 1.5 cm recurrence with direct proximity to left portal vein and bile duct
21.10.15	2	12/8	2/1	New/new	II/II	Subphrenic/subcapsular	15	None	3	–	
10.06.16	1	7	1	New	III	–	12	None	3	–	
31.01.18	2	24/23	2/2	Recurrence/recurrence	VII/VII	Direct proximity V. cava inf./subcapsular	16	None	9	–	Ablation of tumor thrombus in V. cava inf. (4 × 2.5 cm; 2 needles) and intraoperative anticoagulation with 2500 IE heparin
21.03.18	1	10	1	Recurrence	IVa, IVb	Gallbladder bed	5	None	4	–	Ablation of residual tumor thrombus in V. cava inf. (2 cm; 1 needle) and intraoperative anticoagulation with 2500 IE heparin

**Fig. 3:**
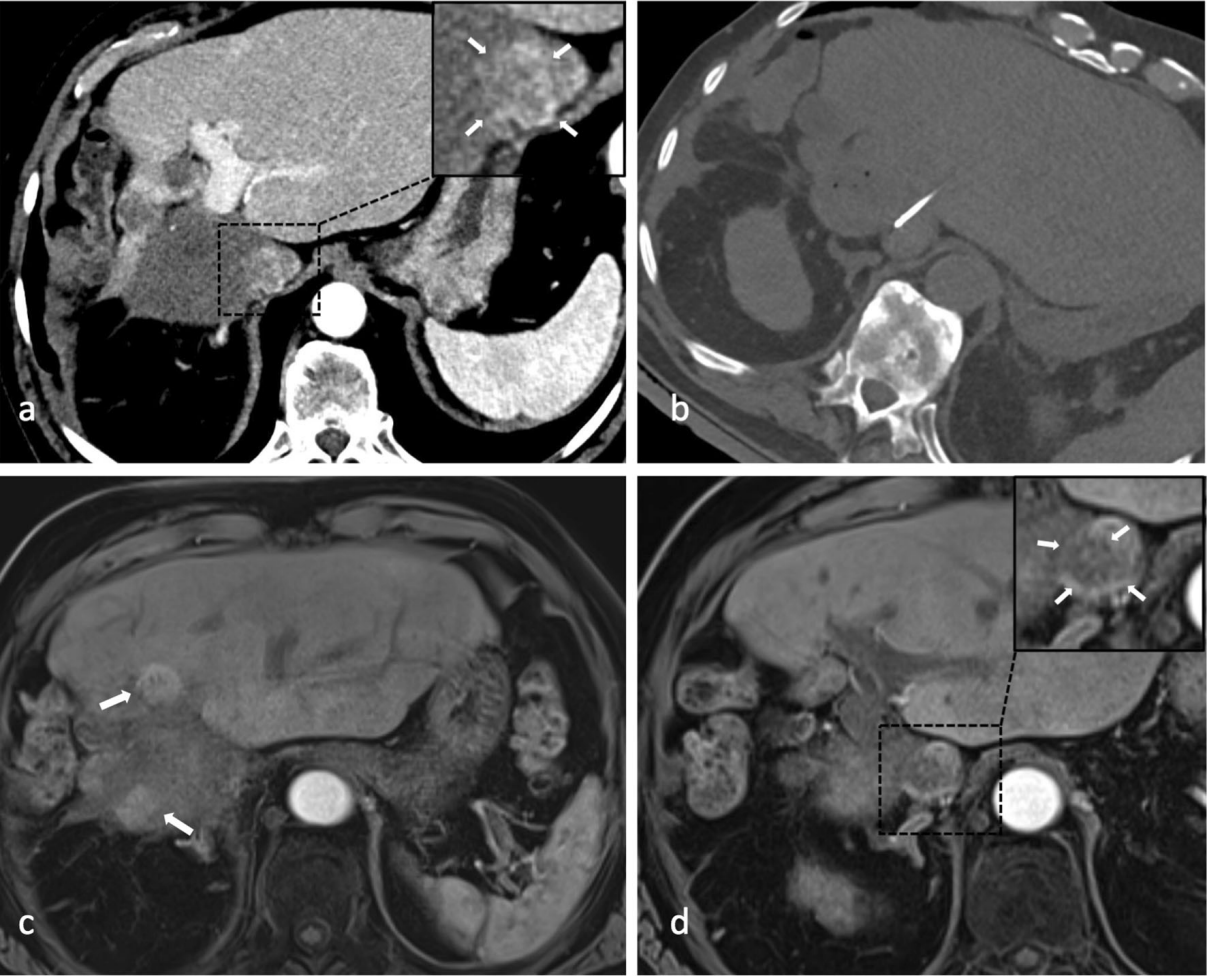
A Arterial phase CT showing contrast enhancing tumor thrombus (magnified image in the right upper corner with white arrows) in January 2018. **B** Axial non-enhanced control CT depicting needle placement for ablation of residual tumor thrombus in inferior caval vein (March 2018); last intervention up until now. **C, D** MRI scan of last follow-up in June 2019 with two recurrent nodules adjacent to necrosis zone (white arrows in **c**) and partially devascularized residual tumor thrombus in inferior caval vein (magnified image in the right upper corner with white arrows in **D**)

Prior to the ninth SRFA session in January 2018, the patient presented with reduced physical fitness, leg edema and weight gain of 4 kg due to an inferior vena cava syndrome (IVCS) caused by a 4 × 2.5 cm measuring tumor thrombus (Fig. 3A). The patient’s case was discussed in a multidisciplinary tumor board consisting of hepatologists, oncologists, transplant surgeons and interventional radiologists where the decision to perform the last two interventions (i.e., January and March 2018) to treat the tumor thrombus as well as the recurrent nodules was made. Unfortunately, CT scans in June 2018 revealed a partial residual tumor thrombus and two intrahepatic recurrent nodules measuring 1.7 cm. Following intense discussion in the multidisciplinary tumor board, a watch-and-wait approach was adopted. No further SRFA treatments have been performed ever since then. A follow-up MRI scan in June 2019 showed one slightly progressive nodule up to 2.3 cm, while the remaining findings appeared unaltered (Fig. 3C, D).

Biochemically, the patient experienced temporal increases of liver enzymes (GOT, GPT, GGT) following the ablations, which were paralleled by an acute phase reaction as illustrated by elevated C-reactive protein levels. Yet, both changes were mitigated within several days without any additional treatment being required. One year after the last intervention (i.e., June 2019), the patient’s liver function was not substantially impaired as illustrated by albumin and bilirubin levels within the physiological range (4126 mg/dl and 0.41 mg/dl, respectively), as well as normal coagulation parameters (i.e., INR of 1.1). In total, 17% of liver volume (April 2007 = 2247 ml; March 2018 = 1865 ml) had been lost over the years.

Except for the sixth session in February 2015, where an additional SIRE was performed, and the first SRFA session, where a post-ablation syndrome occurred, the length of hospital stay did not exceed nine days (mean hospitalization = 4.4 days.) Neither immediate, nor late major complications were noted.

To evaluate the patient’s quality of life, the standardized questionnaire of the World Health Organization (WHOQOL) was utilized and filled out by the patient at each intervention. The patient specified his health status as poor only prior to the first treatment. Procedure-related pain was generally mild (i.e., 3 out of 10 points on a pain scale) and resolved within several days after each intervention. Moreover, the patient did not require external support to master his daily-life activities, suggesting that his general health condition was mostly preserved.

## Discussion

At present, effective approaches for treating unresectable, intrahepatic cholangiocellular carcinoma (ICC) are lacking. Transarterial chemoembolization (TACE) and different systemic therapies have reportedly achieved median overall survival rates of 11.7 and 15.2 months, respectively [4, 5]. In contrast, SRFA represents a potentially curative treatment option with a technical efficacy rate of 92%, yielding an estimated median overall survival of 60 months in unresectable ICC [6], although the sample size of the cited study was small (*n* = 11). With this case report, we reinforce the hypothesis that SRFA represents a promising strategy for treating advanced stages of ICC. In spite of the several limitations of the current report owing to the nature of the study design, our findings have some important implications. First, it appears feasible that SRFA could contribute to substantially prolonging the overall survival of affected individuals. Second, healthy tissue is mostly spared by the procedure, thereby preserving organ function. Third, periods of hospitalization are generally short in patients undergoing SRFA, which contributes to improving their quality of life, and fourth, complications are rare, while procedure-related pain is generally mild. Further investigations are warranted.
